# Research on Recognition Method of Driving Fatigue State Based on Sample Entropy and Kernel Principal Component Analysis

**DOI:** 10.3390/e20090701

**Published:** 2018-09-13

**Authors:** Beige Ye, Taorong Qiu, Xiaoming Bai, Ping Liu

**Affiliations:** Department of Computer, Nanchang University, Nanchang 330029, China

**Keywords:** driving fatigue, sample entropy, kernel principal component analysis, support vector machine

## Abstract

In view of the nonlinear characteristics of electroencephalography (EEG) signals collected in the driving fatigue state recognition research and the issue that the recognition accuracy of the driving fatigue state recognition method based on EEG is still unsatisfactory, this paper proposes a driving fatigue recognition method based on sample entropy (SE) and kernel principal component analysis (KPCA), which combines the advantage of the high recognition accuracy of sample entropy and the advantages of KPCA in dimensionality reduction for nonlinear principal components and the strong non-linear processing capability. By using support vector machine (SVM) classifier, the proposed method (called SE_KPCA) is tested on the EEG data, and compared with those based on fuzzy entropy (FE), combination entropy (CE), three kinds of entropies including SE, FE and CE that merged with KPCA. Experiment results show that the method is effective.

## 1. Introduction

Driving fatigue is a phenomenon in which, due to continuous driving, drivers’ ability of perception, judgment and operation appear to decrease [1]. Drivers are prone to driving fatigue after long driving, and if they keep driving, their limbs will be stiff, their attention will decrease and their judgment will decline. Driving fatigue may cause people to become delirious, and they may be prone to traffic accidents [2]. Therefore, an effective driving fatigue state recognition method is the key to construct the dangerous driving state warning system.

At present, a series of studies has been conducted on the recognition of driving fatigue status at home and abroad. Guo et al. [3] explored the correlation between ECG indicators and driving fatigue state based on ECG signals and constructed the driving fatigue state recognition model combined with the SVM classifier. Yang et al. [4] conducted research on driving fatigue recognition on the basis of the fusion of eye movement and pulse information. Zhao et al. [5] applied functional brain networks to establish a fatigue recognition model based on EEG data and graph theory methods. Zhao et al. [6] constructed a driving fatigue recognition model based on the human eye feature by using a concatenated convolutional neural network. To judge whether the driver felt fatigue, Zhang et al. [7] conducted research on driving fatigue recognition on the feature extraction of the wavelet entropy of EEG signals. Moreover, Chai and Naik et al. [8] used entropy rate bound minimization as a source separation technique, the autoregressive (AR) modeling as the feature extraction algorithm and the Bayesian neural network as the classification algorithm for driving fatigue recognition; they combined independent component by entropy rate bound minimization analysis (ICA-ERBM) and EEG feature extraction components, which have not been explored previously for fatigue classification. Zeng et al. [9] proposed to use deep convolutional neural networks and deep residual learning to predict the drivers’ mental states from EEG signals; they also developed two mental state classification models called EEG-Conv and EEG-Conv-R. Chai and Ling et al. [10] combined the AR modeling feature extractor with a sparse-DBN classifier to constructed a driving fatigue recognition model, which have not been explored previously for EEG-based driving fatigue classification. Hu et al. [11] conducted a driving fatigue recognition model by the combination of the feature set that consisted of sample entropy, fuzzy entropy, approximate entropy and spectral entropy and the gradient boosting decision tree (GBDT) through the use of three different classifiers.

The literature mentioned above has enriched and extended the research of fatigue recognition from different perspectives. EEG signals, with the highest sensitivity in driving fatigue detection and recognition and the highly correlated relationship with the driver’s mental state, have been studied more deeply [12]. There are many ways to analyze EEG signals, such as time domain analysis, frequency domain analysis, multidimensional statistical analysis and nonlinear analysis [13]. However, the recognition accuracy of the driving fatigue state obtained by using these methods is still not satisfactory after the feature extraction of EEG signals. PCA and SVM were used to obtain better recognition accuracy based on motion imagination EEG signals in [14]. However, because of the nonlinear characteristics of EEG data, the internal model of the PCA is linear, the same with the relationship among the principal components. PCA will lose its effectiveness when the principal components of the study object are nonlinear. Therefore, this paper proposes a driving fatigue recognition method based on sample entropy and kernel principal component analysis. There are several advantages to using the sample entropy kernel principal component analysis (SE_KPCA) method. On the one hand, the recognition of driving fatigue state based on sample entropy (SE) [15] is more accurate. On the other hand, the result of dimension reduction in KPCA [16] is more positive and has a strong non-linear processing capability. On the basis of the above, a driving fatigue state recognition model is constructed with the combination of the support vector machine (SVM) [17] algorithm to achieve effective recognition of the driver’s fatigue state.

## 2. Sample Entropy

The sample entropy (SE) calculation process is described as follows, given the original signal of length *N*, denote it by x(1),x(2),…,x(N), and define the *m*-dimensional vector:

(1) $$X_{m}\left(i\right)=\left\{x(i),x(i+1),\ldots ,x(i+m-1)\right\};1\leq i\leq N-m+1$$

Calculate any two *m*-dimensional vectors:

(2) $$D\left[X_{m}\left(i\right),X_{m}\left(j\right)\right]=max\left[x\left(i+k\right)-x\left(j+k\right)\right],0\leq k\leq m-1;i\neq j,\;\;i,j\leq N-m+1$$

$D[X_{m}\left(i\right),X_{m}\left(j\right)]$ is the maximum difference between $X_{m}\left(i\right)$ and $X_{m}\left(j\right)$. Given a threshold *r*, calculate the total number of the maximum difference between any two elements that is less than the threshold:

(3) $$C=\sum_{i=1}^{N-m}\left(D\left(i\right)<r\right)$$

Define a ratio:

(4) $$B_{i}^{m}\left(r\right)=\frac{C}{N-m}$$

$B_{i}^{m}\left(r\right)$ is the ratio of *C* to the total; calculate its mean:

(5) $${\bar{B}}^{m}\left(r\right)=\frac{1}{N-m+1}\sum_{i=1}^{N-m+1}B_{i}^{m}\left(r\right)$$

where ${\bar{B}}^{m}\left(r\right)$ is the proportion mean of the *m*-dimensional sequence. When the signal increases to m+1-dimension, repeat Equation (1) to Equation (4), and calculate the proportion mean of the m+1-dimensional sequence:

(6) $$A^{m+1}\left(r\right)=\frac{C}{N-m}\sum_{i=1}^{N-m}B_{i}^{m+1}\left(r\right)$$

Get the sample entropy of the sequence:

(7) $$SanmEn\left(m,r\right)=\lim_{N\Rightarrow \infty }\left\{-ln(A^{m+1}\left(r\right)/{\bar{B}}^{m}\left(r\right))\right\}$$

When *N* is finite, Equation (7) can be expressed as follows:

(8) $$SanmEn\left(m,r,N\right)=-ln(A^{m+1}\left(r\right)/{\bar{B}}^{m}\left(r\right))$$

From Equation (8), it is known that the value of SanmEn is related to *m* and *r*. Pincus [18] pointed out that the value of *m* is generally taken as two, when *r* is set to be 0.1- to 0.25-times the standard deviation of the original EEG signal time series (0.1 to 0.25 SD; SD is the standard deviation). Thus, *m* is set as two, and *r* is set as 0.25 SD in this paper.

## 3. Principal Component Analysis and Kernel Principal Component Analysis

### 3.1. Basic Principles of PCA

Suppose that the *m*-times extracted data matrix of *n* variables $X_{i},X_{2},\ldots ,X_{m}$ is $X={\left(X_{pq}\right)}_{m*n}$. The main steps of PCA analysis [19,20] are as follows:

1. Calculate the sample mean and standard deviation for each indicator *X*:
  (9) $$\bar{X}=\frac{1}{n}\sum_{p=1}^{m}X_{pq},\;\;S_{q}=\sqrt[{2}]{\frac{1}{N-1}\sum_{p=1}^{m}{\left(X_{pq}-\bar{X_{q}}\right)}^{2}},\;\;q=1,2,\ldots ,n$$
2. Normalize $X_{pq}$ and calculate its normalization matrix:
  (10) $$Y_{pq}=\frac{X_{pq}-\bar{X_{q}}}{m},\;\;p=1,2,\ldots ,m,\;\;\;q=1,2,\ldots ,n$$
3. Calculate the correlation coefficient matrix *R* according to the obtained standardized matrix $Y={\left(Y_{pq}\right)}_{m*n}$:
  (11) $$r_{qk}=\frac{1}{m-1}\sum_{p=1}^{m}Y_{pq}*Y_{pk}$$
  (12) $$R={\left(r_{pq}\right)}_{mn},\;\;r_{qq}=1,\;\;r_{qk}=r_{kq}$$
4. Get the eigenvalue of *R*, denoted as λ. Suppose $\lambda_{1}\geq \lambda_{2}\geq \ldots \geq \lambda_{n}>0$ and $l_{1},l_{2},\ldots ,l_{n}$ are the corresponding feature vectors. Determine the range of *K* according to the cumulative variance contribution as CVC > 90%, and define the CVC as:
  (13) $$CVC=\sum_{q=1}^{k}\lambda_{q}/\sum_{q=1}^{n}\lambda_{q}$$
  the *K* principal components are created, denoted as:
  (14) $$Z_{q}=l_{q}X$$

### 3.2. Basic Principles of KPCA

There are *M* samples in the input space, denoted as $x_{k}\left(k=1,2,\ldots ,M\right),x_{k}\in R^{N},\sum_{k=1}^{M}x_{k}=0$. The nonlinear mapping function Φ is introduced to the algorithm, transforming the sample points in the input space $x_{1},x_{2},\ldots ,x_{M}$ into sample points in the feature space as $\Phi \left(x_{1}\right),\Phi \left(x_{2}\right),\ldots ,\Phi \left(x_{M}\right)$, and the hypothesis:

(15) $$\sum_{k=1}^{M}\Phi \left(x_{k}\right)=0$$

Then, the covariance matrix in the feature space *F* is defined as:

(16) $$\bar{C}=\frac{1}{M}\sum_{j=1}^{M}\Phi \left(x_{j}\right)\Phi {\left(x_{j}\right)}^{T}$$

Therefore, the solving equation of PCA in the feature space is:

(17) $$\lambda V=\bar{C}v$$

λ is the eigenvalue, and $v\in F\backslash \left\{0\right\}$ is the eigenvector, so:

(18) $$\lambda \left(\Phi \left(x_{k}\right)*v\right)=\Phi \left(x_{k}\right)*\bar{C}v,\left(k=1,2,\ldots ,M\right)$$

Note that *v* can be expressed linearly by $\Phi \left(x_{i}\right)\left(i=1,2,\ldots ,M\right)$ in the above formula.

(19) $$v=\sum_{i=1}^{M}a_{i}\Phi \left(x_{i}\right)$$

where $a_{1},a_{2},\ldots ,a_{N}$ is constant. Define an N∗N matrix satisfying the Mercer condition, denoted as *K*:

(20) $$K_{ij}=\Phi \left(x_{i}\right)*\Phi \left(x_{j}\right)$$

*K* is called the nuclear matrix, which can be obtained from Equation (16) to Equation (19) as follows:

(21) Mλa=Ka

The required eigenvalues and eigenvectors are obtained by solving the formula Equation (21). The projection of the test sample on the *F*-space vector $V^{k}$ is:

(22) $$\left(V^{k}*\Phi \left(x\right)\right)=\sum_{i=1}^{M}a_{i}^{k}\left(\Phi \left(x_{i}\right)*\Phi \left(x\right)\right)$$

Supposed that Equation (15) is not valid. Then, the *K* in Equation (21) is replaced by $\tilde{K}$.

(23) $$\tilde{K_{ij}}=K_{ij}-\frac{1}{M}\sum_{m=1}^{M}l_{im}K_{mj}-\frac{1}{M}\sum_{n=1}^{M}K_{in}l_{nj}+\frac{1}{M^{2}}\sum_{m,n=1}^{M}l_{im}K_{mn}l_{nj}$$

where $l_{ij}=1$ (for all *i*, *j*).

### 3.3. Kernel Function Methods

At present, there are several forms of kernel functions that can be chosen, as follows:

1. Linear kernel function (special case):
  (24) $$K\left(x,x_{i}\right)=x*x_{i}$$
2. *P*-order polynomial kernel function:
  (25) $$K\left(x,x_{i}\right)={\left[\left(x*x_{i}\right)+1\right]}^{p}$$
3. Radial basis function (RBF):
  (26) $$K\left(x,x_{i}\right)=exp\left(-\frac{||x-x_{i}\left|\right|}{\delta^{2}}\right)$$
4. Multilayer perceptual (MLP) kernel function:
  (27) $$K\left(x,x_{i}\right)=tanh\left[v\left(x*x_{i}\right)+c\right]$$

The *P*-order polynomial kernel function, radial basis function and multilayer perceptual kernel function are used in the model of this paper.

## 4. EEG Data Processing Method Based on Sample Entropy and Principal Component Analysis/Kernel Principal Component Analysis

### 4.1. EEG Data Processing Method Based on Sample Entropy and Principal Component Analysis

Based on the above discussion about the method proposed in this paper, the algorithm that combined sample entropy and principal component analysis (SE_PCA) can be divided into the following three steps:

1. Collect the EEG signal, and preprocess it; then, extract the sample entropy characteristics of the data by the formula Equations (1) to (8), and obtain a matrix $X_{m*n}$;
2. Take the matrix $X_{m*n}$ into the formula Equations (9) to (14), then calculate its principal component;
3. Construct a model, and use SVM to classify.

### 4.2. EEG Data Processing Method Based on Sample Entropy and Kernel Principal Component Analysis

Based on the above discussion about the method proposed in this paper, the SE_KPCA algorithm can be divided into the following four steps:

1. Collect the EEG signal, and preprocess the EEG signal; then, extract the sample entropy characteristics of the data by the formula Equations (1) to (8), and obtain a matrix $X_{m*n}$;
2. Select the kernel function $K(x,x_{i})$, the matrix $X_{m*n}$ as an input of KPCA, and centralize it in high dimensional space; then, calculate matrix $\tilde{K}$ according to Equation (23);
3. Calculate the eigenvalues and eigenvectors of the matrix $\tilde{K}$, as well as its nonlinear principal component;
4. Construct a model, and use SVM to classify.

## 5. Method Testing and Result Analysis

### 5.1. Test Environment and Test Data

Test environment: The platform environment used in the experiment includes a static simulator (Beijing-China Joint Teaching Equipment Co., Ltd., ZY-31D vehicle driving simulator, Beijing, China), and this includes three 24-inch monitors and a software teaching system for driving simulations (ZM-601 V9.2). A 32-electrode EEG collecting cap, the computer system (windows 10 × 64), EEG collecting and preprocessing software (Neuroscan 3.2) and EEG analysis software (MATLAB R2014b) were used.

**Figure 1:**
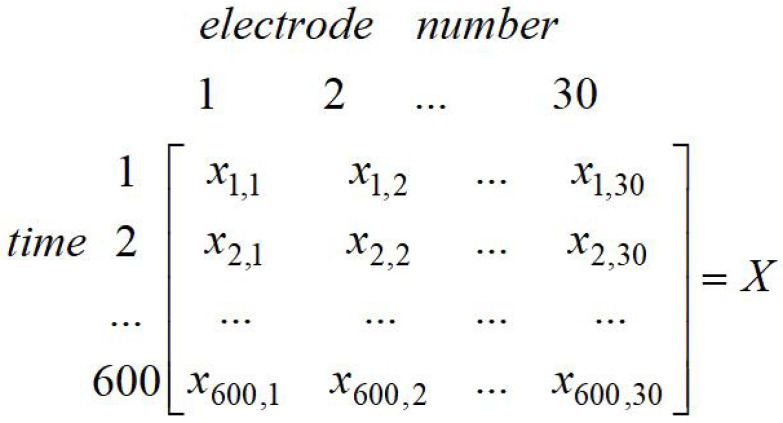
Sample entropy data matrix (10 individuals).

**Figure 2:**
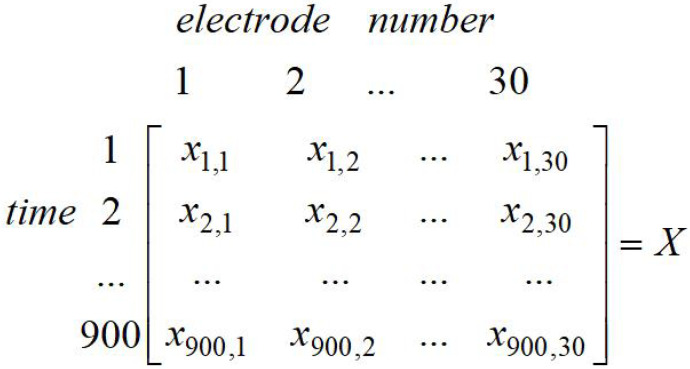
Sample entropy data matrix (15 individuals).

Test data description: The EEG signal data analyzed in this paper come from the EEG study, which simulated car driving training. Twenty five normal subjects were tested for the current fatigue level during the training, such as the sleep quality on the previous night, the diet on the day, etc., then two sets of experiment data were recorded by every subject, namely fatigue state and non-fatigue state. According to the precious experience in the fatigue-related experiment, each subject was asked to drive for 40 min without a break, then they were asked to take a questionnaire to check their states [21]. The EEG data are 32-electrode, 600 s time series at a sampling rate of 1000 Hz, which consisted of 300 s of rest (non-fatigue) and 300 s of fatigue. After collecting a person’s EEG signal data, filtering and processing them (artifact removal, removal of eye movement interference, signal correction, etc. [21]) were conducted. This paper conducted two sets of experiments. The first one was taking some data from 10 individuals and 60 s for each person (the first 30 s in the non-fatigue state and the other 30 s in the fatigue state), which constructed a 600 * 30 data matrix (for which 600 is 600 s, 30 is the 30 electrodes), as shown in Figure 1. The other one was taking some data from 15 individuals and 60 s of each person, which constructed a 900 * 30 data matrix, as shown in Figure 2. At present, this paper merely compares the experimental results of 30 s, due to less data being able to reduce the time of experiment and the amount of data in 30 s being enough. However, the subsequent experiments would enlarge the selection of different time bands for testing.

### 5.2. Driving Fatigue State Recognition Test Based on SE_PCA

Firstly, the SE_PCA method was used to analyze the contribution ratio of the 30 electrode principal components. According to formula $\sum_{q=1}^{n}\lambda_{q}$ mentioned in Section 3.1, the contribution rate of the 30 electrode principal components was calculated as shown in Table 1, in which *i* represents the principal components (or principal elements) and $C_{i}$ represents the contribution rate. From Table 1, each principal element corresponds to two sets of contribution rate, and the former data are from the first experiment and the latter from the second experiment. In the first experiment, the cumulative contribution rate of the top 10 principal components reached 90.63%. As a result, the amount of principal components was reduced from 30 to 10. Similarly, the cumulative contribution rate of the top 14 principal components reached 95.08%, and the cumulative contribution rate of the top 23 principal components reached 99.10%. In the second experiment, the cumulative contribution rate of the top eight principal components reached 90.14%. As a result, the amount of principal components was reduced from 30 to eight. Similarly, the cumulative contribution rate of the top 13 principal components reached 95.17%, and the cumulative contribution rate of the top 23 principal components reached 99.12%.

Secondly, we selected the main component through Equation (13) >90%. This article mainly tests three kinds of situations where the contribution rates reach 90%, 95% and 99%, respectively. The corresponding characteristic variables in the three cases were 10, 14 and 23 in the first experiment; the corresponding characteristic variables in the three cases were 8, 13 and 23 in the second experiment.

Finally, according to the three contribution rates, the accuracy of the recognition in driving fatigue was tested by the SVM classifier. This paper used a method based on *k*-fold cross-validation in which k=3. Seventy percent of the data were used as a training set, then the other thirty percent of the data were used as a test set. The test results are shown in Table 2 and Table 3. Compared with the driving fatigue recognition accuracy rates, which only used the sample entropy, when the contribution rate reached 0.99, the SE_KPCA method improved the recognition accuracy of the driving fatigue state compared with the SE method, and the time performance had also been reduced.

### 5.3. Driving Fatigue State Recognition Test Based on SE_KPCA

First of all, we analyzed the principal components contribution rates by the SE_KPCA method. For example, when the kernel function chose a *P*-order polynomial kernel function and the parameter was set as P=2, the calculation results were as shown in Table 4. As we can see, each principal element corresponds to two contribution rates; the former data were from the first experiment, and the latter data were from the second experiment, the same for Table 5 and Table 6.

Then, we selected the main component, and the cumulative contribution rate calculation was consistent with the PCA method. For example, the kernel function chose a *P*-order polynomial kernel function testing for three cases with contribution rates of 90%, 95% and 99%. In the first experiment, Table 4 shows the result when parameter P=2, and the characteristic variables in the three cases were: 8, 12, 26. Table 5 shows the result when parameter P=1, and the characteristic variables in the three cases were: 9, 14, 23. Table 6 shows the result when parameter P=0.5, and the characteristic variables in the three cases were: 10, 14, 22. For the second experiment, Table 4 shows the result when parameter P=2, and the characteristic variables in the three cases were: 7, 12, 25. Table 5 shows the result when parameter P=1, and the characteristic variables in the three cases were: 8, 13, 23. Table 6 shows the result when parameter P=0.5, and the characteristic variables in the three cases were: 9, 13, 21.

Last but not least, the three selected principal components for driving fatigue recognition accuracy were tested by the SVM classifier. The specific test was performed under three different principal component contribution rates by using the KPCA method of the *P*-order polynomial kernel function, radial basis function and multilayer perceptual kernel function, and every optimal parameters was obtained through multiple experiments. The test results are shown in Table 7, Table 8 and Table 9. The data test results of 15 people are shown in Table 10, Table 11 and Table 12. These tables also include the accuracy of the driving fatigue state recognition based on SE_PCA under the same contribution rates.

The test results from Table 7, Table 8, Table 9, Table 10, Table 11 and Table 12 show that the SE_KPCA method was better than the SE_PCA method at identifying and classifying driving fatigue. In particular, when KPCA’s kernel functions chose a radial basis function with a parameter of 0.2 and a contribution rate of 0.9, the classification accuracy of the SE_KPCA method reached 98.33%, and the time performance was good. The subsequent experiments in this paper all used the radial basis function, and the parameter σ was 0.2, while the contribution rate was set as 0.9.

### 5.4. Comparison Test between SE_KPCA and the Driving Fatigue Recognition Method Based on Fuzzy Entropy/Combination Entropy

**Figure 3:**
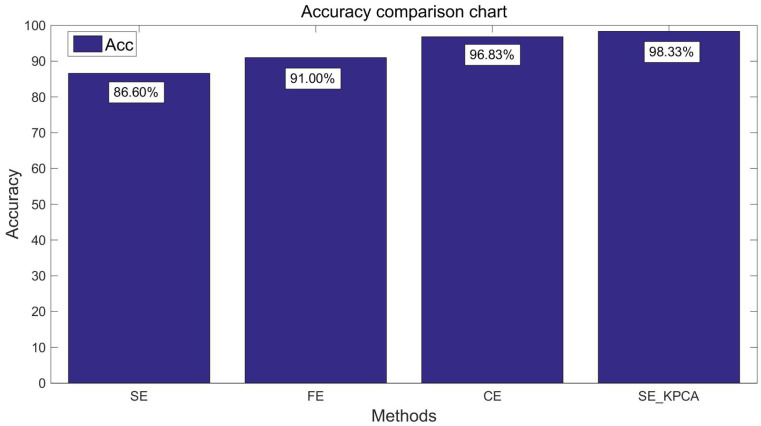
Comparison of the recognition accuracy rates among the sample entropy, fuzzy entropy, combination entropy and SE_KPCA methods (10 individuals).

**Figure 4:**
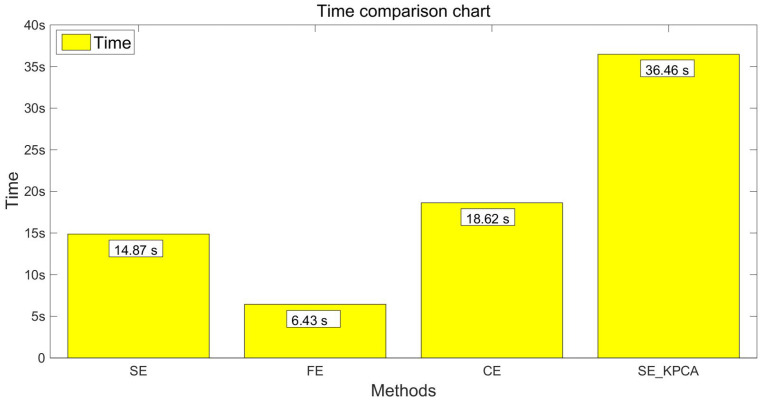
Comparison of the time among the sample entropy, fuzzy entropy, combination entropy and SE_KPCA methods (10 individuals).

**Figure 5:**
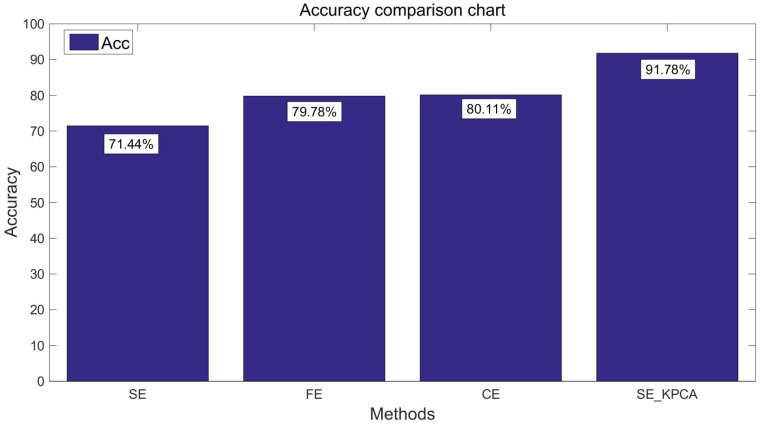
Comparison of the recognition accuracy rates among the sample entropy, fuzzy entropy, combination entropy and SE_KPCA methods (15 individuals).

**Figure 6:**
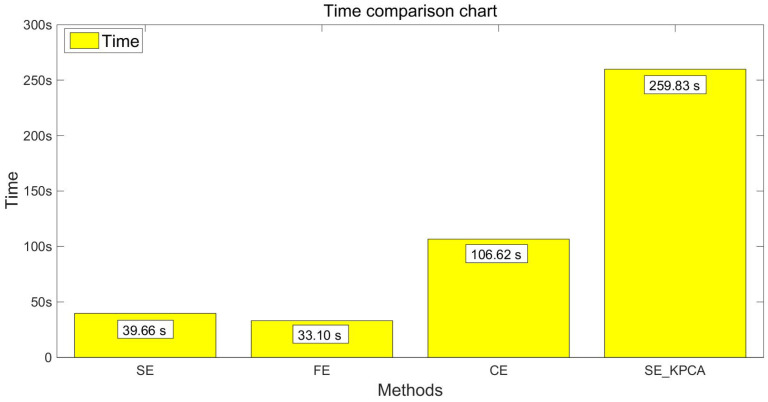
Comparison of the time among the sample entropy, fuzzy entropy, combination entropy and SE_KPCA methods (15 individuals).

In order to verify the classification effect of the SE_KPCA method further, traditional methods of feature extraction were used to compare the sample entropy, fuzzy entropy (FE) [13,22] and combination entropy (CE) [16]. Take the samples of 10 and 15 individuals’ EEG signals as an example; fuzzy entropy and combination entropy were used for feature extraction, and then SVM was applied to identify the driving fatigue state; the test results are shown from Figure 3, Figure 4, Figure 5 and Figure 6. After the comparison and analysis of the figure, the conclusion was draw that SE_KPCA had significantly improved the classification recognition rate compared with the traditional sample entropy, fuzzy entropy and combination entropy, and the time performance was good.

### 5.5. Comparison of the SE_KPCA Method Based on KPCA and Fuzzy Entropy/Combination Entropy for Driving Fatigue Identification

#### 5.5.1. Data Description

(1) The EEG data processing method based on KPCA and fuzzy entropy (FE_KPCA):

- Extract the features of fuzzy entropy from the collected EEG signals according to the fuzzy entropy formula in the literature [22];
- Select the kernel function $K(x,x_{i})$; centralize the fuzzy entropy data in the high dimensional space, and then, calculate the matrix according to Equation (23);
- Calculate the eigenvalues and eigenvectors of the matrix $\tilde{K}$;
- Calculate its nonlinear principal component.

(2) The EEG data processing method based on the KPCA and combination entropy (CE_KPCA):

- Extract the features of the combination entropy from the collected EEG signals according to the combination entropy formula in the literature [21];
- Select the kernel function $K(x,x_{i})$; centralize the combination entropy data in the high dimensional space, and then, calculate the matrix according to Equation (23);
- Calculate the eigenvalues and eigenvectors of the matrix $\tilde{K}$;
- Calculate its nonlinear principal component.

#### 5.5.2. Experimental Results

**Figure 7:**
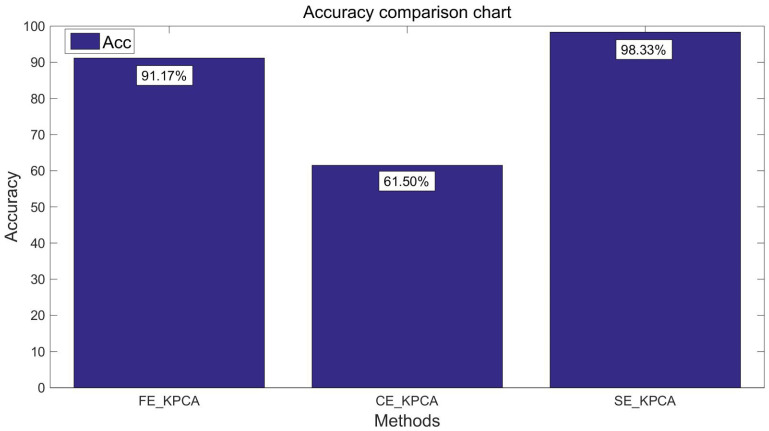
Comparison of the recognition accuracy rates among the fuzzy entropy KPCA (FE_KPCA), combination entropy KPCA (CE_KPCA) and SE_KPCA methods (10 individuals).

**Figure 8:**
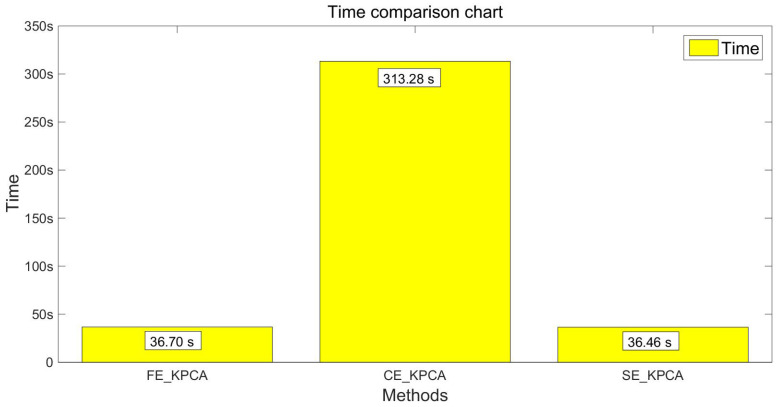
Comparison of the time among the FE_KPCA, CE_KPCA and SE_KPCA methods (10 individuals).

**Figure 9:**
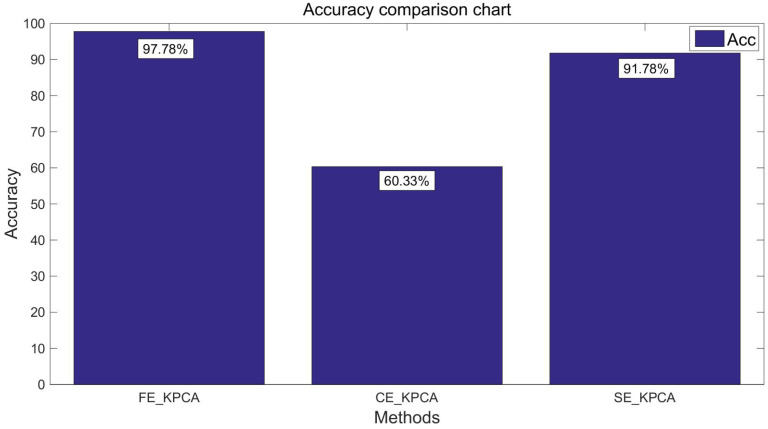
Comparison of the recognition accuracy rates among the FE_KPCA, CE_KPCA and SE_KPCA methods (15 individuals).

**Figure 10:**
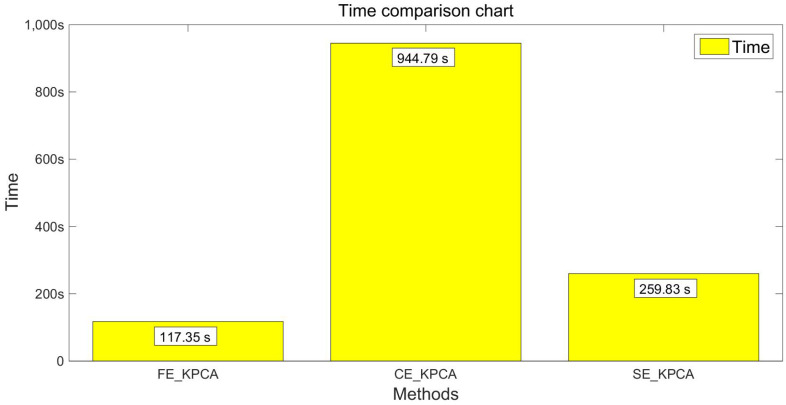
Comparison of the time among the FE_KPCA, CE_KPCA and SE_KPCA methods (15 individuals).

After verifying the validity of the SE_KPCA method in Section 5.4, this paper compares KPCA combined with fuzzy entropy (FE_KPCA) with KPCA combined with combination entropy (CE_KPCA). As shown from Figure 7, Figure 8, Figure 9 and Figure 10, through the same method, the SVM was adopted for classification and identification. After comparing and analyzing all the figures, our conclusion is that the classification recognition rate of SE_KPCA was obviously higher than FE_KPCA and CE_KPCA, and the temporal performance was lower.

As shown from Figure 3, Figure 4, Figure 5, Figure 6, Figure 7, Figure 8, Figure 9 and Figure 10, it can also be seen that FE_KPCA and CE_KPCA have no higher classification accuracy than traditional FE and CE, and the time performance is worse.

## 6. Conclusions

This paper studies the characteristics of EEG signals in two groups (fatigue state and non-fatigue state). Firstly, feature extraction of the EEG signal was conducted by applying sample entropy, then further feature extraction was made by using kernel principal component analysis, and the SVM classifier was used to classify and identify the two states of fatigue and non-fatigue. Through analysis and comparison of the experiment results that indicate when the kernel functions select the radial basis function, the classification recognition rate performs excellently. Besides, when compared with the traditional methods, the classification recognition rate is also significantly improved. This paper mainly researched the experiment results of the entropy, PCA and KPCA, but the subsequent experiments will introduce more methods for testing.

## Figures and Tables

**Table 1:** Contribution rates of each principal component.

*i*	$C_{i}$	*i*	$C_{i}$	*i*	$C_{i}$	*i*	$C_{i}$	*i*	$C_{i}$
1	0.5664	7	0.0275	13	0.0097	19	0.0043	25	0.0016
	0.6286		0.0197		0.0068		0.0033		0.0017
2	0.0721	8	0.0209	14	0.0086	20	0.0038	26	0.0013
	0.0960		0.0163		0.0060		0.0030		0.0014
3	0.0633	9	0.0191	15	0.0079	21	0.0031	27	0.0012
	0.0470		0.0145		0.0056		0.0028		0.0012
4	0.0486	10	0.0154	16	0.0065	22	0.0027	28	0.0011
	0.0350		0.0107		0.0050		0.0027		0.0009
5	0.0425	11	0.0138	17	0.0051	23	0.0023	29	0.0011
	0.0322		0.0104		0.0046		0.0024		0.0008
6	0.0306	12	0.0125	18	0.0044	24	0.0018	30	0.0008
	0.0266		0.0080		0.0040		0.0019		0.0007

**Table 2:** Comparison of the sample entropy principal component analysis (SE_PCA) and SE methods (10 individuals).

Contribution Rate	SE_PCA		SE	
	Acc	Time	Acc	Time
0.90	80.50%	7.68 s		
0.95	81.83%	8.25 s	86.60%	14.87 s
0.99	88.00%	10.34 s		

**Table 3:** Comparison of the SE_PCA and SE methods (15 individuals).

Contribution Rate	SE_PCA		SE	
	Acc	Time	Acc	Time
0.90	59.00%	20.18 s		
0.95	66.33%	27.64 s	71.44%	39.66 s
0.99	73.78%	35.95 s		

**Table 4:** Contribution rates of each principal component of the *P*-order (P=2) polynomial kernel function.

*i*	$C_{i}$	*i*	$C_{i}$	*i*	$C_{i}$	*i*	$C_{i}$	*i*	$C_{i}$
1	0.7026	7	0.0190	13	0.0059	19	0.0027	25	0.0013
	0.7171		0.0156		0.0052		0.0024		0.0013
2	0.0534	8	0.0157	14	0.0057	20	0.0023	26	0.0013
	0.0679		0.0131		0.0042		0.0023		0.0011
3	0.0412	9	0.0141	15	0.0048	21	0.0019	27	0.0011
	0.0359		0.0118		0.0037		0.0020		0.0009
4	0.0318	10	0.0093	16	0.0037	22	0.0018	28	0.0010
	0.0313		0.0089		0.0034		0.0019		0.0009
5	0.0269	11	0.0077	17	0.0034	23	0.0016	29	0.0009
	0.0214		0.0070		0.0030		0.0017		0.0008
6	0.0213	12	0.0073	18	0.0030	24	0.0015	30	0.0008
	0.0183		0.0064		0.0028		0.0016		0.0006

**Table 5:** Contribution rates of each principal component of the *P*-order (P=1) polynomial kernel function.

*i*	$C_{i}$	*i*	$C_{i}$	*i*	$C_{i}$	*i*	$C_{i}$	*i*	$C_{i}$
1	0.5977	7	0.0257	13	0.0087	19	0.0034	25	0.0017
	0.6255		0.0205		0.0066		0.0033		0.0016
2	0.0809	8	0.0208	14	0.0079	20	0.0030	26	0.0016
	0.1035		0.0177		0.0055		0.0031		0.0013
3	0.0557	9	0.0197	15	0.0065	21	0.0026	27	0.0013
	0.0456		0.0139		0.0046		0.0026		0.0012
4	0.0434	10	0.0127	16	0.0051	22	0.0022	28	0.0011
	0.0390		0.0112		0.0046		0.0025		0.0009
5	0.0355	11	0.0104	17	0.0044	23	0.0021	29	0.0010
	0.0288		0.0091		0.0042		0.0023		0.0008
6	0.0284	12	0.0097	18	0.0041	24	0.0019	30	0.0009
	0.0255		0.0076		0.0042		0.0021		0.0006

**Table 6:** Contribution rates of each principal component of the *P*-order (P=0.5) polynomial kernel function.

*i*	$C_{i}$	*i*	$C_{i}$	*i*	$C_{i}$	*i*	$C_{i}$	*i*	$C_{i}$
1	0.5055	7	0.0314	13	0.0107	19	0.0041	25	0.0020
	0.5343		0.0249		0.0081		0.0041		0.0020
2	0.1051	8	0.0250	14	0.0097	20	0.0036	26	0.0019
	0.1374		0.0224		0.0069		0.0039		0.0016
3	0.0685	9	0.0238	15	0.0079	21	0.0032	27	0.0015
	0.0562		0.0159		0.0061		0.0033		0.0014
4	0.0552	10	0.0157	16	0.0062	22	0.0027	28	0.0013
	0.0476		0.0135		0.0058		0.0030		0.0011
5	0.0424	11	0.0128	17	0.0054	23	0.0025	29	0.0012
	0.0366		0.0112		0.0052		0.0028		0.0009
6	0.0353	12	0.0128	18	0.0051	24	0.0023	30	0.0010
	0.0330		0.0089		0.0051		0.0026		0.0007

**Table 7:** Comparison between the sample entropy kernel principal component analysis (SE_KPCA) (P-order) method and the SE_PCA method (10 individuals).

Contribution Rate	SE_KPCA				SE_PCA
	Parameter	P=2	P=1	P=0.5	
0.90	Acc	74.50%	73.83%	73.33%	80.50%
	Time	66.08 s	14.74 s	6.41 s	7.68 s
0.95	Acc	82.5%	82.33%	75.83%	81.83%
	Time	83.25 s	15.70 s	7.40 s	8.25 s
0.99	Acc	93.17%	85.83%	75.83%	88.00%
	Time	99.63 s	17.77 s	10.36 s	10.34 s

**Table 8:** Comparison between the SE_KPCA (RBF) method and the SE_PCA method (10 individuals).

Contribution Rate	SE_KPCA					SE_PCA
	Parameter	σ=0.1	σ=0.2	σ=0.7	σ=1	
0.90	Acc	93.80%	98.33%	89.50%	81.80%	80.50%
	Time	91.60 s	36.46 s	7.58 s	6.47 s	7.68 s
0.95	Acc	93.80%	98.33%	92.60%	85.80%	81.83%
	Time	116.19 s	45.00 s	13.05 s	8.57 s	8.25 s
0.99	Acc	93.80%	98.33%	92.80%	86.30%	88.00%
	Time	138.92 s	54.01 s	28.50 s	21.51 s	10.34 s

**Table 9:** Comparison between the SE_KPCA (MPL) method and the SE_PCA method (10 individuals).

Contribution Rate	SE_KPCA					SE_PCA
	Parameter	c=0.1	c=0.2	c=0.7	c=1	
		v=0.001	v=0.01	v=0.001	v=0.01	
0.90	ACC	70.60%	70.30%	70.67%	70.50%	80.50%
	Time	4.03 s	3.83 s	3.97 s	3.81 s	7.68 s
0.95	ACC	79.10%	79.60%	79.10%	73.80%	81.83%
	Time	14.15 s	13.82 s	14.05 s	13.48 s	8.25 s
0.99	ACC	88.00%	86.33%	88.00%	85.83%	88.00%
	Time	7.09 s	7.10 s	7.07 s	6.80 s	10.34 s

**Table 10:** Comparison between the SE_KPCA (P-order) method and the SE_PCA method (15 individuals).

Contribution Rate	SE_KPCA				SE_PCA
	Parameter	P=2	P=1	P=0.5	
0.90	Acc	58.56%	56.89%	57.89%	59.00%
	Time	235.78 s	40.49 s	19.09 s	20.18 s
0.95	Acc	66.78%	66.22%	57.223%	66.33%
	Time	268.56 s	43.84	22.24 s	27.64 s
0.99	Acc	75.56%	72.11%	58.56%	73.78%
	Time	325.98 s	65.78 s	35.64 s	35.95 s

**Table 11:** Comparison between the SE_KPCA (RBF) method and the SE_PCA method (15 individuals).

Contribution Rate	SE_KPCA					SE_PCA
	Parameter	σ=0.1	σ=0.2	σ=0.7	σ=1	
0.90	Acc	91.44%	91.78%	80.89%	66.11%	59.00%
	Time	411.60 s	259.83 s	50.80 s	41.20 s	20.18 s
0.95	Acc	91.44%	91.67%	83.89%	74.33%	66.33%
	Time	416.19 s	361.75 s	72.98 s	43.39 s	27.64 s
0.99	Acc	91.56%	91.67%	84.44%	75.89%	73.78%
	Time	458.92 s	395.39 s	239.60 s	99.86 s	35.95 s

**Table 12:** Comparison between the SE_KPCA (MPL) method and the SE_PCA method (15 individuals).

Contribution Rate	SE_KPCA					SE_PCA
	Parameter	c=0.1	c=0.2	c=0.7	c=1	
		v=0.001	v=0.01	v=0.001	v=0.01	
0.90	ACC	41.11%	40.78%	41.11%	40.33%	59.00%
	Time	12.52 s	11.91 s	11.22 s	11.94 s	20.18 s
0.95	ACC	62.78%	61.78%	62.78%	61.33%	66.33%
	Time	14.15 s	13.82 s	14.05 s	13.48 s	27.64 s
0.99	ACC	73.11%	71.78%	73.11%	71.56%	73.78%
	Time	22.12 s	21.26 s	22.13 s	21.19 s	35.95 s
